# Sentence comprehension test for Russian: A tool to assess syntactic competence

**DOI:** 10.3389/fpsyg.2023.1035961

**Published:** 2023-02-10

**Authors:** Daria Chernova, Artem Novozhilov, Natalia Slioussar

**Affiliations:** ^1^Institute for Cognitive Studies, Saint Petersburg University, Saint Petersburg, Russia; ^2^School of Linguistics, HSE University, Moscow, Russia

**Keywords:** sentence comprehension, syntactic processing, grammatically complex sentences, reading, listening, working memory, Russian language

## Abstract

Although all healthy adults have advanced syntactic processing abilities in their native language, psycholinguistic studies report extensive variation among them. However, very few tests were developed to assess this variation, presumably, because when adult native speakers *focus* on syntactic processing, *not being distracted* by other tasks, they usually reach ceiling performance. We developed a Sentence Comprehension Test for the Russian language aimed to fill this gap. The test captures variation among participants and does not show ceiling effects. The Sentence Comprehension Test includes 60 unambiguous grammatically complex sentences and 40 control sentences that are of the same length, but are syntactically simpler. Every sentence is accompanied by a comprehension question targeting potential syntactic processing problems and interpretation errors associated with them. Grammatically complex sentences were selected on the basis of the previous literature and then tested in a pilot study. As a result, six constructions that trigger the largest number of errors were identified. For these constructions, we also analyzed which ones are associated with the longest word-by-word reading times, question answering times and the highest error rates. These differences point to different sources of syntactic processing difficulties and can be relied upon in subsequent studies. We conducted two experiments to validate the final version of the test. Getting similar results in two independent experiments, as well as in two presentation modes (reading and listening modes are compared in Experiment 2) confirms its reliability. In Experiment 1, we also showed that the results of the test correlate with the scores in the verbal working memory span test.

## 1. Introduction

The ability to process syntactically complex sentences efficiently is a crucial skill for text comprehension. Although any adult native speaker without language disorders has this ability, numerous psycholinguistic studies in the last decades demonstrate considerable by-subject variability in performance on syntactic processing tasks [see (Farmer et al., 2012) for an overview]. The assessment of syntactic processing abilities is included in many clinical tests in various languages (e.g., Cho-Reyes and Thompson, 2012; Mack et al., 2016; Frizelle et al., 2018; Akinina et al., 2021), as well as in many first and second language acquisition tests (Wiig et al., 2013; Montgomery et al., 2016; Lopukhina et al., 2019; Vernice et al., 2019), and neurotypical adult native speakers are used in these tests as a control group. However, very few tests aim to study variation within this group.

Only two studies on English, by Acheson et al. (2008) and by Dąbrowska (2018), pursued this goal (Dąbrowska also included non-native participants). As far as we can judge, the reason is as follows: when healthy adult native speakers focus on syntactic processing, their performance reaches the ceiling level. As a result, the majority of sentence-level processing studies with such participants pick out one or two particular constructions and explore the implications of their complexity for different parsing models.

We believe that an instrument to assess sentence comprehension in healthy adult native speakers is necessary. It could be used in a battery of tests targeting variation in language skills along with vocabulary tests (e.g., Nation and Beglar, 2007; Lemhöfer and Broersma, 2011), tests of word reading efficiency (e.g., Torgesen et al., 2012), spelling tests (e.g., Andrews et al., 2020), tests assessing a general print exposure like the author recognition test (e.g., Moore and Gordon, 2015) etc. Such test batteries are used in a variety of psycholinguistic and neurolinguistic projects: for example, in the Multilingual Eye-tracking Corpus (MECO) project that aimed to study the role of individual differences in various language-related skills when reading in typologically different languages (Siegelman et al., 2022). So far, there were no syntactic processing tests to include in these batteries.

In this paper, we develop a Sentence Comprehension Test (SCT) for the Russian language. We conducted a pilot study to identify syntactic constructions that are non-trivial to process. Then we validated the test in Experiments 1 and 2. We showed that it captures by-subject variability and does not induce ceiling effects. Getting similar results in two independent experiments, as well as in two presentation modes (reading and listening modes are compared in Experiment 2) confirms the validity of the SCT. As we show in section 1.2, by-subject variability in syntactic processing skills is usually associated with the differences in the working memory span. In Experiment 1, we additionally demonstrated that the results of the SCT correlate with the scores in a working memory test. We also analyzed differences between the constructions selected for the SCT: word-by-word reading times, comprehension question answering times and accuracy. This information can be used in subsequent studies focusing on syntactic processing difficulties in Russian.

The structure of the paper is as follows. In section 1.1, we discuss the two existing tests of syntactic processing skills, Acheson et al. (2008) and Dąbrowska (2018), as well as some relevant experimental studies conducted on Russian. In Section 1.2, we present a brief overview of verbal working memory tests and discuss their relevance for syntactic processing. In Section 1.3, we discuss whether any differences can be expected in syntactic processing in the reading and listening modality. In Section 2, we go over various constructions that have been described as syntactically complex in the previous literature and were selected for the pilot and final version of our Sentence Comprehension Test. After that, a pilot study and two experiments aimed to validate the final version of the test are presented.

### 1.1. Existing tests assessing syntactic processing

Only two tests assessing syntactic processing in healthy adult native speakers have been developed so far, and both are for the English language. The first study was by Acheson et al. (2008). The sentences in their Sentence Comprehension Task were shown word by word in a self-paced reading mode and followed by questions with two response options: “yes” or “no.” Reading speed and answer accuracy were measured.

The test included five types of constructions: sentential complements, subject and object relative clauses, extended subordinate clauses, and sentences with multiple prepositional phrases. Some questions were designed to assess the comprehension of complex syntax (for example, *The witness that the investigator contacted waited outside the small café.* —*Did the investigator contact the witness*?), the others were not (for example, *Although the potatoes were shredded very carefully by the assistant cook, they came out unevenly and were unattractive.* —*Were the potatoes shredded carelessly*?). All participants performed relatively well, which brings us back to the problem of ceiling accuracy in syntactic processing tasks.

Acheson et al. (2008) were interested in the relationship between syntactic processing skills and print exposure, which was assessed using updated versions of the Author Recognition Test and the Magazine Recognition Test (Stanovich and West, 1989), and several questionnaires. No correlation was found. However, Acheson and colleagues observed that both accuracy and reading speed significantly correlated with the scores on the verbal portion of the American College Test, a standardized achievement test. The authors concluded that there is little evidence directly linking print exposure and sentence-level processing, suggesting that individual differences in this domain are more likely to be explained by working memory span variability.

Another test to assess syntactic aspects of sentence comprehension was developed by Dąbrowska (2018). Her Pictures and Sentences Test was based on several previous studies (Dąbrowska and Street, 2006; Wells et al., 2009; Street and Dąbrowska, 2010). It included ten types of constructions: active and passive semantically reversible sentences, subject and object clefts, subject and object relatives, locative constructions with or without quantifiers, possessive locative constructions with quantifiers (for example, *Every table has a lamp on it*) and sentences with subjects modified by prepositional phrases.

Four of the constructions (actives, simple locatives, subject relatives and subject clefts) were selected as syntactically simple, while the other six constructions were expected to be more challenging. Simple constructions were included in the test as control conditions. The participants were asked to read sentences one by one and to select matching pictures. For example, after the sentence *It was the girl that the man fed* they had to select between a picture where a man was feeding a girl and a picture where a girl was feeding a man.

Both native English speakers and second language (L2) learners were tested. Native speakers exhibited ceiling performance on the four control conditions, which shows that they had understood the task and were cooperative. At the same time, experimental sentences showed considerable by-subject variability. It was found that the grammar comprehension correlates with vocabulary size, level of education, print exposure and understanding of collocations.

No tests targeting healthy adult native speakers have been developed for other languages, including Russian. Nevertheless, Malyutina et al. (2018) designed a sentence comprehension task for their study of age-related differences in syntactic processing in Russian. А set of 100 sentences was constructed: 74 grammatically complex sentences and 26 grammatically simpler sentences. Grammatically complex sentences belonged to several types which are known to cause processing difficulties: constructions with embedded sentences modifying a complex noun phrase (attached to its head or to the dependent noun in genitive case), semantically reversible sentences with the noncanonical object–verb–subject and the canonical subject–verb–object word order; subject and object relative clauses and sentences with reflexive pronouns. Every sentence was followed by a comprehension question with two response options.

Two experimental sessions were done: a self-paced reading session and a session where the presentation rate was twice as fast as the participant’s average reading speed measured during the first session. The authors showed that answering accuracy was affected by age and presentation rate, but found no interaction between these factors. The average accuracy rate was 0.75–0.80, but by-subject and by-type variability, as well as the problem of ceiling effects, were not discussed in this study.

### 1.2. Verbal working memory and sentence comprehension

Numerous studies show that working memory is interconnected with a number of linguistic abilities (e.g., Just and Carpenter, 1992; Schwering and MacDonald, 2020).^1^ Some studies focus on language production (e.g., Daneman and Green, 1986; Acheson and MacDonald, 2009), the others on language comprehension, especially on syntactic processing (e.g., Daneman and Carpenter, 1980; King and Just, 1991; MacDonald et al., 1992; Caplan and Waters, 1999, 2013; Swets et al., 2008; Caplan et al., 2013). Correlations with vocabulary size and the level of language anticipation have also been reported (Farmer et al., 2012). Therefore, we may expect that the results of a valid sentence comprehension test would correlate with a verbal working memory test and included such test in our study.

There are several tests for verbal working memory span assessment, including the alphabet span task (Craik, 1986), the backward digit span task (Botwinick and Storandt, 1974), various n-back tasks etc. However, the most widely used verbal working memory test was designed by Daneman and Carpenter (1980). In this test, participants are presented with increasingly longer sequences of sentences: two, three, four or five sentences in a sequence. They either listen to them or read them (silently or aloud) and then are asked to recall the final words of each sentence in the exact form. It has been shown that both reading and listening versions of this test significantly correlate with several measures of reading comprehension, like fact retrieval and pronominal reference, while the digit span and word span tests do not (Daneman and Carpenter, 1980). For this reason we decided to use the Russian adaptation of this test developed by Fedorova (2003) in the present study.

### 1.3. The written or oral mode of presentation in sentence comprehension

The question whether listening comprehension is more or less costly than reading comprehension is controversial. On the one hand, ontogenetically, reading skills are acquired later than oral speech. Many authors assume that while reading, we activate not only orthographic, but also phonological representations (Frost, 1998). Moreover, while listening we can rely prosodic cues that help us to analyze the syntactic structure of the sentence and to resolve ambiguity. Evidence from language pathology shows that people with aphasia generally experience more difficulties with reading than with listening (DeDe, 2012). On the other hand, the reader can regulate the processing pace spending more time on the fragments that are more difficult to process, while the listener cannot. In most languages, segmenting continuous speech into words, one of the earliest crucial processing steps, does not rely on any obvious unambiguous clues, while in written texts word boundaries are clearly demarcated in many modern languages.

Several studies tried to find out experimentally which modality is more difficult, and at least for syntax-oriented tasks like grammaticality judgment the results are controversial. Vetter et al. (1979) compared visual and auditory presentation, normal or monotone, as well as simultaneous visual and auditory presentation, and found no overall effect of modality. Murphy (1997) showed that participants were slower and less accurate in grammaticality judgments about oral stimuli compared to written ones.

However, no study suggested that some syntactic constructions would be more difficult in one modality, while the others in the other. The same grammatical system is used in both modalities. In the neurocognitive research, there is evidence for a supramodal language system that integrates linguistic input from speech to print and activates a common code (Shankweiler et al., 2008; Braze et al., 2011). Certain brain areas, like the interferior frontal gyrus, the middle and superior temporal gyri and the angular gyrus, show modulation of activity depending on sentence type regardless of the presentation mode (Constable et al., 2004).

Therefore, we might expect parallel findings in the oral and written version of the Sentence Comprehension Test we created. Replicating the most important results in both modalities would confirm the reliability of the test. Moreover, this would give more freedom to its potential users.

## 2. Selecting syntactically complex sentences

To create a Sentence Comprehension Test for Russian, we identified several constructions that had been shown to cause processing difficulties in the previous experimental studies. As we show in more detail below, these difficulties may have different sources. Some of them are purely syntactic, like the presence and number of embedded clauses in the sentence or a noncanonical word order. These two factors are mentioned in many studies, and Boyle et al. (2013) even assess their relative significance in triggering processing difficulties in English speaking children (and confirm that noncanonical word orders are especially difficult to process). In morphologically rich languages, morphosyntax may cause processing problems: for example, Russian speakers often fail to track case features to decide on the syntactic structure of certain constructions (Chernova, 2015; Chernova et al., 2016).

Processing difficulties may also arise at the intersection between syntax and semantics. For example, according to the embodied cognition approach, language comprehension involves mental simulation of the situation (Fischer and Zwaan, 2008). As a result, it is more difficult to comprehend sentences where the order of mention does not coincide with the chronological order of the events (Opačić and Osgood, 1984) or where the objects are mentioned not in the order they are manipulated (Dragoy et al., 2015). Our aim in this study was not to focus on one or two sources of processing difficulties, but to include as many diverse constructions as we could, so that our test covered a representative range of them. Henceforth, we will use the cover term *syntactically complex sentences* to refer to the selected constructions, keeping in mind that the nature of their complexity may be different.

The pilot version of the test included ten types of target constructions listed below, eight sentences per each type, as well as 50 grammatically simple control sentences. After 30 participants completed the pilot version, we selected six constructions (types I–V and VII in the list below) that caused significantly more comprehension errors than control sentences. The final version of the SCT included these six constructions, 10 sentences per each type, as well as 40 control sentences. This version was tested in two experiments.

Comparing the SCT with the tests by Acheson et al. (2008) and Dąbrowska (2018), only two constructions in the pilot SCT coincided with the ones used in these papers, and only one of them (object relative clauses) was included in the final version. While the English tests used yes–no questions or pictures to assess sentence comprehension, we created a question with a choice of two answers for every target or control sentence. An example is given in (1a,b).^2^

(1)  a. *Konvert peredali pomoščniku detektiva, sledivšemu za podozrevaemym.*
    envelope_ACC_ gave_PL_ assistant_DAT_ detective_GEN_ pursuing_DAT_ after suspect_INS_
    ‘They gave the envelope to the assistant_i_ of the detective_j_ (who was) pursuing_i_ the suspect.’
 b. Question:
    *Kto sledil za podozrevaemym*?
    who pursued after suspect_INS_
    *‘*Who was pursuing the suspect?’
    Response options:
    A) detektiv B) pomoščnik
     detective assistant
     ‘the detective’ ‘the assistant’

All test sentences were unambiguous, so only one response was correct. To make the task non-trivial, both response options were always mentioned in the sentence. Moreover, we aimed to make all sentences semantically reversible and unbiased, i.e., the two response options referred to equally plausible scenarios in order to exclude guessing. Thus, the syntactic structure of the sentence had to be analyzed to give a correct answer, it could not be chosen based on plausibility considerations.

Now let us go over different syntactic constructions selected for the pilot and final versions of the SCT.

### 2.1. Types I and II: Sentences with high and low attachment of a participial modifier (HA and LA)

Structures with modifier attachment ambiguity, as in (2), have been extensively studied in the processing literature (Frazier, 1979; Cuetos and Mitchell, 1988; Grillo and Costa, 2014 etc.). In these structures, a modifier — a relative clause, a participial clause, or a PP — can be attached either to the head of a complex noun phrase (high attachment, or HA) or to the dependent noun (low attachment, or LA). Cross-linguistic research demonstrated that when other factors are balanced, some languages prefer HA and the other LA.

(2) *the maid of the actress that was on the balcony* /*standing on the balcony/with red hair.*

In Russian, some sentences are ambiguous, like (2), while in the others, the attachment site is morphologically disambiguated: by the number or gender of the *wh*-word heading the relative clause or by the number, gender or case of the participle.^3^ Chernova (2015), Chernova et al. (2016) studied examples with participial modifiers and showed that readers make a lot of interpretation errors with disambiguation by case: in the sentences like (1) with HA and especially like (3) with LA. These constructions were also included in the sentence comprehension task by Malyutina et al. (2018).

(3)  a. *Notarius napisal nasledniku millionera, živšego za granicej.*
    notary_NOM_ wrote heir_DAT_ millionaire_GEN_ living_GEN_ across border_INS_
    ‘The notary wrote to the heir_i_ of the millionaire_j_ living_j_ abroad.’
 b. Question: Who lived abroad?
    Response options: A) the millionaire; B) the heir

From Chernova (2015); Chernova et al. (2016) and from further research by Slioussar et al. (2022) we know that error rates are much lower in the examples disambiguated by number- and gender-specific endings. We also know that while the readers eventually prefer HA (making fewer interpretation errors in unambiguous HA sentences and selecting HA interpretations more often in ambiguous ones), LA is easier to process online, i.e., unambiguous LA sentences are read faster than HA ones. Discussing the implications of this finding is beyond the scope of this paper, so let us only make one observation that is important for the current study. This means that the readers do not fail to notice some of the relevant features in online processing.^4^ However, they often fail to retrieve case features when deciding on the overall syntactic structure of the sentence in the offline task, i.e., answering interpretation questions [see Slioussar et al., 2022 for a possible explanation].

### 2.2. Type III: Temporal constructions

Clark and Clark (1968) were the first to study the processing of English sentences with four temporal conjunctions: *after, before, and then, but first*. They found that native speakers made much fewer mistakes with the *before* sentences than with the *after* sentences (subordinate clauses always followed matrix clauses in their study). This could be explained by the fact that in the *before* sentences, the order of the events corresponds to the order in which they are mentioned. Subsequently, numerous studies investigated the processing of these constructions in special populations: children (Clark, 1971; Townsend and Ravelo, 1980), patients with aphasia (Sasanuma and Kamio, 1976), and patients with mental disorders (Natsopoulos and Xeromeritou, 1988; Natsopoulos et al., 1991).

Fedorova (2005) studied the processing of similar constructions in Russian. Two factors were manipulated: the order of the matrix and subordinate clauses and the conjunction used (meaning ‘before’ or ‘after’). The results of the study were different from English: it showed that sentences where the matrix clause comes first, and sentences with ‘before’ cause more processing difficulties. The source of these differences still has to be explained, but for the present study, a more general observation is important: sentences with temporal conjunctions like (4a) may cause processing difficulties when the order of the events is to be established.

(4)  a. *Pered tem kak Tolja propylesosit pol, Julja vyguljaet sobaku.*
    before Tolja_NOM_ will-vacuum-clean_3SG_ floor_ACC_ Julia_NOM_ will-walk_3SG_ dog_ACC_
    ‘Before Tolja vacuum cleans the floor, Julia will walk the dog.’
 b. Question: What happens first?
    Response options: A) Tolja vacuum cleans the floor; B) Julja walks the dog

### 2.3. Type IV: Spatial constructions

Spatial constructions of the type ‘A under B’, ‘A behind B’ etc. are known to be especially challenging for patients with semantic aphasia (Luria, 1970; Dragoy et al., 2015) and children (Statnikov and Akhutina, 2013). Laurinavichyute et al. (2017) investigated how adult native speakers without neurological impairments process spatial structures like (5) depending on the word order and sensorimotor stereotypes that reflect normal sequences of object manipulation. Their eye-tracking study using the visual word paradigm found roughly the same high accuracy rates in the conditions that matched or mismatched sensorimotor stereotypes. Nevertheless, we hypothesized that reversible constructions of this kind can pose at least some comprehension difficulties if they include both a mismatched sensorimotor stereotype and a noncanonical word order (with the prepositional phrase preceding the direct object).

(5)  a. *Passažir sprjatal v seryj jaščik kožanyj čemodan.*
    passenger_NOM_ hid_SG_ into gray box_ACC_ leather suitcase_ACC_
    ‘The passenger hid the leather suitcase in the gray box.’
 b. Question: What was hidden where?
    Response options: A) the box in the suitcase; B) the suitcase in the box.

*Type V. Complex comparative sentences.* Reversible comparative constructions like (6) have been shown to be especially difficult for patients with semantic aphasia (Luria, 1970; Akhutina, 2016; Dragoy et al., 2015). These constructions are assumed to incur additional processing costs because the order of the objects according to the scope of comparison does not correspond to the order in which they are mentioned in the sentence.

(6)  a. *Šerstjanaja jubka dlinnee šelkovoj, no koroče l’njanoj.*
    woolen_NOM_ skirt_NOM_ longer silk_INS_ but shorter linen_INS_
    ‘The woolen skirt is longer than the silk one, but shorter than the linen one.’
 b. Question: Which skirt is longer?
    Response options: A) the silk one; B) the linen one.

### 2.4. Types VI and VII: Sentences with a subject relative clause or an object relative clause (SRC and ORC)

Subject relative clauses (SRC), like in (7), and especially object relative clauses (ORC), like in (8), were shown to be associated with a considerable working memory load (King and Just, 1991). Both examples include subordinate clauses, and in ORCs, a noncanonical word order is an additional source of complexity. According to Ferreira (2003), various constructions with noncanonical word order (ORCs, passives,^5^ object clefts) often cause comprehension difficulties and misinterpretation. Processing complexity of Russian relative clauses was discussed in several studies (Levy et al., 2013; Price and Witzel, 2017). Examples with SRCs and ORCs were included in the sentence comprehension task by Malyutina et al. (2018) and in the sentence processing task for children with and without developmental disorders (Rakhlin et al., 2016).

(7)  a. *Starik, kotoryj v polnoč ožidal na kladbišče kolduna, nepodvižno stojal*
    old-man_NOM_, who_NOM_ at midnight_ACC_ waited_SG_ at cemetery_LOC_ sorcerer_GEN_ motionless stood_SG_ *u ogrady.* at fence_GEN._
    ‘The old man, who was waiting for the sorcerer at the cemetery at midnight, stood motionless at the fence.’
 b. Question: Who stood at fence?
    Response options: A) the old man; B) the sorcerer

(8)  a. *Svidetel’, kotorogo upomjanul v svoej reči istec, vskočil so svoego* *mesta.*
    witness_NOM,_ whom_ACC_ mentioned_SG_ in his_LOC_ speech_LOC_ claimant_NOM,_ jumped_SG_ from his_GEN_ seat_GEN_
    ‘The witness whom the claimant mentioned in his speech jumped up from his seat.’
 b. Question: Who was mentioned?
    Response options: A) the witness; B) the claimant.

### 2.5. Type VIII: Sentences with a noncanonical OVS word order

The object–verb–subject (OVS) sentences^6^ may trigger processing difficulties not only due to their noncanonical word order, but also due to their contextual requirements. While the canonical SVO order is felicitous in isolation, other orders have contextual requirements in terms of information structure. For example, the OVS order is felicitous when the object is given and the subject is new. If noncanonical orders are used in isolation, they are associated with additional processing costs (Sekerina, 2003; Slioussar, 2011). Malyutina et al. (2018) included semantically reversible OVS sentences like (9) in their sentence processing task.

(9)  a. *Na polovine puti oxotnika izdaleka gromko okliknul lesnik.*
    on half_LOC_ way_GEN_ hunter_ACC_ from-afar loudly hailed_SG_ forester_NOM_.
    ‘Halfway through the trip, the hunter was loudly hailed from afar by a forester.’
 b. Question: Who shouted loudly?
    Response options: A) the forester B) the hunter.

### 2.6. Type IX: Constructions with a genitive NP

Semantically reversible constructions with a noun modified by another noun phrase in the genitive case (10) have been shown to cause processing difficulties in Russian speaking patients with semantic aphasia (Luria, 1970). The source of these difficulties is morphosyntactic: the failure to track word order and case features to build the right syntactic structure.

(10)  a. *Na ploščadke ja vstretil brata moego druga s bol’šoj sobakoj.*
    at playground_LOC_ I_NOM_ met_SG_ brother_ACC_ my_GEN_ friend_GEN_ with big_INS_ dog_INS_
    ‘At the playground I met my friend’s brother with a big dog.’
 b. Question: Who did he meet?
    Response options: A) his brother’s friend; B) his friend’s brother

### 2.7. Type X: Sentences with conversives

We also included constructions with antonyms and conversives (11), which entail a mutual change of the roles of the agent and the patient and therefore can be potentially confusing. Here the source of potential problems is semantic rather than syntactic, so in hindsight, these examples were not fully suitable for our test. They also did not cause significant processing problems in the pilot study, so we did not include them in the final version for both reasons.

(11)  a. *Posle prazdnikov sniženie cen značitel’no uveličilo prodaži.*
    after holidays_GEN_ decline_NOM_ prices_GEN_ significantly increased_SG_ sales_ACC_
    ‘After the holidays the decrease of the prices significantly increased the sales.’
 b. Question: What happened with the sales?
    Response options: A) they have decreased; B) they have increased.

## 3. Pilot study

We conducted a pilot study to find out which of the constructions we selected cause significant comprehension problems.

### 3.1. Participants

Thirty native speakers of Russian (17 female) aged 18–32 volunteered to take part in the pilot study. In all experiments reported in this paper, the participants were not informed about the purpose of the study. All experiments were carried out in accordance with the Declaration of Helsinki and existing Russian and international regulations concerning ethics in research. All participants provided informed consent.

### 3.2. Materials

The pilot study included the ten types of constructions listed in section 2 (8 examples per type) and 50 control sentences. Thus, there were 130 sentences in total followed by comprehension questions with a choice of two answers. The sentences were from 5 to 15 words long, control and target sentences had roughly the same average length. However, control sentences did not belong to the types I–X listed above and did not have any other properties described as syntactically complex in the previous literature. An example is given in (12).

(12)  a. *Posle koncerta ballerina pytalas’ najti za kulisami kostjumeršu.*
    after concert_GEN_ ballerina_NOM_ tried_SG_ to-find behind curtains_INS_ costume-designer_ACC_
    ‘After the concert the ballerina tried to find the costume designer behind the curtain.’
 b. Question: Who was looked for?
    Response options: A) ballerina; B) costume designer

Control sentences were introduced for two reasons. Firstly, we were concerned that if all sentences were relatively complex, participants would pay extra attention to their syntactic structure. This would affect the ecological validity of the experiment and might result in participants reading more slowly than they normally would, but making very few errors. So our control sentences served as fillers, masking the aim of the study. Secondly, to argue that wrong answers to target sentences are due to their syntactic complexity, we had to compare them to some relatively simple sentences. If multiple errors were made in all sentences, other explanations would have to be invoked, like the general incooperativeness of the participants.

### 3.3. Procedure

The pilot study was conducted on a web-based platform Ibex Farm (Drummond et al., 2016). Like Acheson et al. (2008), we used the non-cumulative word-by-word self-paced reading paradigm (Just et al., 1982) to make the task less trivial. Each experimental session began with instructions and a consent form. The participants were instructed to read sentences and answer the questions as quickly and accurately as they could. After that the participant saw a sentence on the screen, in which all words were masked by dashes, while spaces and punctuation remained intact. Each time the participant pressed the space bar, a word was revealed, the previous word was re-masked, and reading times were measured. Then a question and two response options appeared. Accuracy and response time were measured. The experiment started with two training sentences, and then target and control sentences followed in a random order.

### 3.4. Analysis

In the pilot study, we analyzed only participants’ accuracy because the number of participants was not sufficient for a reliable analysis of reading time and answering time data. Nevertheless, mean reading and answering times by condition are also reported: in many psycholinguistic studies the average reading speed is considered as one of the main measures of processing difficulty. To calculate the means, RTs that exceeded a threshold of 2.5 standard deviations by condition were excluded (Ratcliff, 1993). In total, 13.6% of the reading time data and 10.1% of the answering time data were excluded.

The statistical analysis was done in the *R* programming environment.^7^ We modeled accuracy data with a mixed-effects logistic regression using the *glmer* function from the *lme4* package (Bates et al., 2015). For *post hoc* analyses, Tukey’s tests were conducted using the *glht* function from the *multcomp* package (Bretz et al., 2010). Random intercepts and random slopes by a participant and by an item were included in the models.

### 3.5. Results and discussion

Average accuracy, word-by-word reading times and answering times for different types of target and control sentences are presented in Table 1.

**Table 1:** Average word-by-word reading times, question answering times and accuracy in the pilot study.

Sentence type	Average accuracy (%)	Number of correct answers per person	Mean reading time (ms)	Mean answering time (ms)
Comparative	88	5–7	652.2	5486.9
Conversive	94	7–8	501.5	3521.9
Genitive	93	6–8	519.6	3462.4
HA	72	4–8	752.2	3990.9
LA	70	2–8	744.5	3991.1
Locative	88	5–8	534.2	4675.2
ORC	87	4–8	597.8	3355.1
OVS	95	7–8	545.6	2616.9
SRC	93	6–8	547.3	3346.0
Temporal	85	4–8	486.6	3754.0
Control	99.5	48–50	513.8	1960.5

In the statistical analysis, the type of the construction was a fixed factor and the comprehension accuracy was a dependent variable; we used Tukey’s test to compare different constructions with each other. Seven types of target sentences caused a significantly larger number of errors than control sentences: types I–V, VII, and IX, i.e., sentences with a HA or LA of a participial clause, with temporal clauses, ORCs, locative, comparative and genitive constructions. The results of the statistical analysis are given in Table 2 (here and below, only significant results are reported for the sake of readability, all results are available at: https://osf.io/a8t7n/). Out of these seven types we selected six types for which participants gave less than 90% correct answers (genitive constructions were excluded) and used them in the final version of the Sentence Comprehension Test. We can also see that word-by-word reading times and answering times tend to be larger for target sentences than for control sentences, so we will analyze these variables in Experiments 1 and 2.

**Table 2:** Significant model outputs for accuracy analysis in the pilot study.

Comparison	Model output
Control vs. Comparative	*β* = −3.03, SE = 0.60, *z* = −5.05, *p* < 0.001***
Genitive vs. Control	*β* = −2.35, SE = 0.62, *z* = −3.76, *p* = 0.008**
HA vs. Conversive	*β* = −2.00, SE = 0.60, *z* = −3.30, *p* = 0.041*
HA vs. Control	*β* = −4.05, SE = 0.56, *z* = −7.31, *p* = <0.001***
LA vs. Conversive	*β* = −2.16, SE = 0.60, *z* = −3.62, *p* = 0.015*
LA vs. Control	*β* = −4.20, SE = 0.55, *z* = −7.61, *p* = <0.001***
LA vs. Genitive	*β* = −1.86, SE = 0.57, *z* = −3.25, *p* = 0.048*
Locative vs. Control	*β* = −2.80, SE = 0.60, *z* = −4.70, *p* = <0.001***
ORC vs. Control	*β* = −3.45, SE = 0.57, *z* = −6.08, *p* = <0.001***
OVS vs. HA	*β* = 2.58, SE = 0.69, *z* = 3.75, *p* = 0.008**
OVS vs. LA	*β* = 2.74, SE = 0.69, *z* = 3.99, *p* = 0.003**
SRC vs. HA	*β* = 2.36, SE = 0.65, *z* = 3.61, *p* = 0.013*
SRC vs. LA	*β* = 2.52, SE = 0.65, *z* = 3.87, *p* = 0.005**
Temporal vs. Control	*β* = −3.18, SE = 0.58, *z* = −5.51, *p* = <0.001***

* indicate results significant at the *p*<0.05 level, ** indicate results significant at the *p*<0.01 level, and *** to indicate results significant at the *p*<0.001 level.

## 4. Experiment 1: A reading study with a working memory span test

The goal of the experiment was to validate the final version of the Sentence Comprehension Test that we constructed taking the results of the pilot study into account. We aimed to show that the SCT captures by-subject variability and does not suffer from ceiling effects. In addition to that, we aimed to check whether the scores of the SCT correlate with the scores of the working memory span test. As we showed in the introduction, working memory span was found to be crucial for syntactic processing and was implicated in many explanations of individual differences.

### 4.1. Participants

Forty-two native speakers of Russian (29 female) aged 19–32 (mean age 23, students of different universities of Saint Petersburg) volunteered to take part in Experiment 1. All of them reported that they had no language disorders or neurological problems. No participant took part in the pilot study.

### 4.2. Materials

The final version of the SCT included 60 target sentences of the six types (10 examples per type): sentences with a HA or LA of a participial clause, ORCs,^8^ temporal clauses, locative and comparative constructions (types I–V and VII presented in section 2). The full list of sentences can be found at: https://osf.io/a8t7n/. In the first three constructions, the sources of potential difficulties are in grammar, being morphosyntactic in HA and LA sentences and syntactic in ORCs. ORCs are assumed to be difficult due to a combination of two syntactic factors: an embedded clause and a noncanonical word order. The pilot study demonstrated that none of these factors alone was sufficient to trigger a significant number of errors. In temporal clauses, as well as in locative and comparative constructions, the main source of potential difficulties is a mismatch between syntax and semantics: the order of mention does not coincide with the chronological order of events in temporal constructions, the objects are mentioned not in the order they are manipulated in locative constructions and not in an ascending or descending order in comparative constructions (in addition to that, temporal sentences include an embedded clause and comparative sentences include two comparative phrases).

We also included 40 control sentences that did not belong to these six types of constructions, but had roughly the same length as target sentences (8.10 and 8.16 words respectively, with the range of 6–12 words). Thus, there were 100 sentences in total followed by comprehension questions with a choice of two answers. To assess the working memory span of the participants we used the test by Daneman and Carpenter (1980) adapted for Russian by Fedorova (2003).

### 4.3. Procedure

For the sentence comprehension task, the procedure was the same as in the pilot study. The working memory test was run online by a video call on Zoom.^9^ The experimenter shared the screen with the participant, on which test sentences were presented one by one. The participant was asked to read them aloud, and the experimenter switched the slide as soon as the participant finished reading a sentence. There were 2–5 sentences in a sequence, after which the participant saw a blank slide and was given 10 s to recall the last word of each sentence (in the exact form). Each correct guess gave the participant a point in the final score. In total, there were 70 sentences.

### 4.4. Analysis

In the Sentence Comprehension Test, we analyzed participants’ word-by-word reading time, question answering time and accuracy. RTs that exceeded a threshold of 2.5 standard deviations by condition were excluded (Ratcliff, 1993). In total, 12.8% of the reading time data and 7.2% of the answering time data were excluded. For the working memory test, we calculated the total sum of correct answers.

The statistical analysis was done in the *R* programming environment,^10^ as in the pilot study. We modeled RT data with a mixed-effects regression using the *lmer* function from the *lme4* package, and accuracy data with a mixed-effects logistic regression using the *glmer* function from the *lme4* package (Bates et al., 2015). To obtain the values of *p* from the *t* values given by the model, we used the *lmerTest* package (Kuznetsova et al., 2017). For *post hoc* analyses, Tukey’s tests were conducted using the *glht* function from the *multcomp* package (Bretz et al., 2010). Random intercepts and random slopes by a participant and by an item were included in the models.

### 4.5. Results and discussion

First of all, we analyzed accuracy in the SCT and found that target sentences were significantly more difficult to process than control sentences (80.6 vs. 92.6% correct answers on average; *β* = 0.25, SE = 0.04, *z* = 6.01, *p* < 0.01). Target sentences also had longer word reading times than controls (on average, 701.9 vs. 673.1 ms, *β* = 38.11, SE = 12.38, *t* = 3.08, *p* < 0.01) and longer question answering times (on average, 3622.3 vs. 3096.6 ms, *β* = 575.53, SE = 140.30, *t* = 4.10, *p* < 0.01). Both measures were assumed to indicate a higher processing load for target sentences.

**Figure 1:**
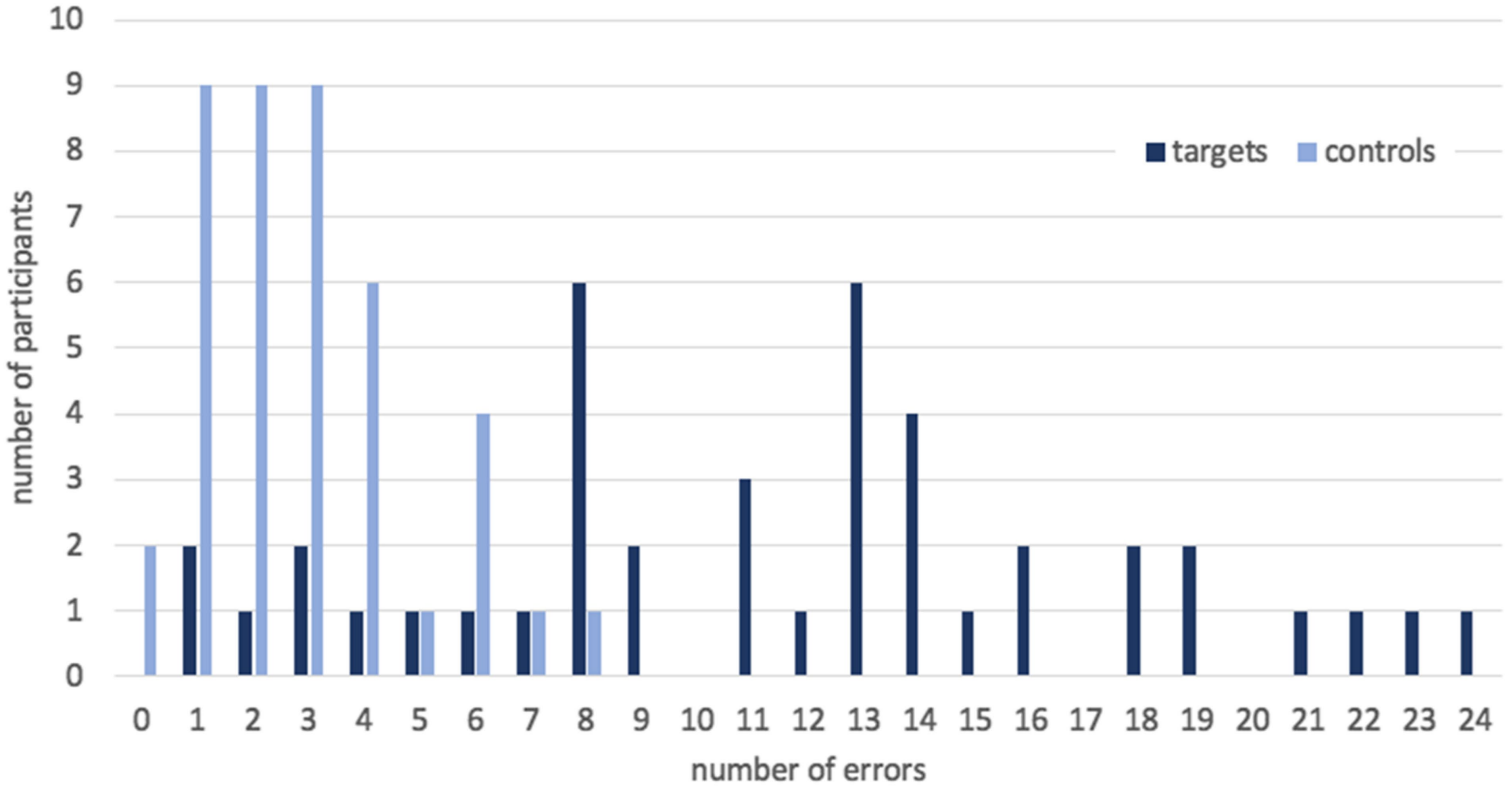
The number of errors per participant in target and control sentences in Experiment 1.

Secondly, we detected an extensive variation in accuracy between participants (see Figure 1). In target sentences, they made from 1 to 24 errors, which means from 98 to 60% correct answers. The mean number of errors was 11.6, SD = 6.0. At the same time, the number of errors in control sentences did not vary that much: from 0 to 8, which means from 100 to 80% correct answers, with three fourths of participants making no more than two errors. The mean number of errors was 2.9, SD = 1.9.

Working memory tests scores also varied a lot: from 26 to 65 out of 70. The mean score was 46.1, SD = 8.1. These scores correlated significantly with accuracy for target sentences (*r* = 0.59, *p* < 0.01) and for control sentences (*r* = 0.52, *p* < 0.01). This shows that the variation we observed is not random and correlates with individual differences in the working memory span, which is consistent with the previous studies discussing by-subject variation in syntactic abilities.

Finally, we were interested in the differences between the target constructions we selected. Average accuracy, word-by-word reading times and question answering times are presented in Table 3. The results of pairwise statistical comparisons of accuracy between different constructions are given in Table 4. Importantly, every target construction was significantly different from control sentences, except for the temporal one. High and low attachment sentences triggered the largest number of errors (74.3 and 62.6% correct responses respectively), comparative sentences were the next (81.9% correct responses). Low attachment sentences were significantly different from all other types except for high attachment and comparatives.

**Table 3:** Average word-by-word reading times, question answering times and accuracy in Experiment 1.

Sentence type	Average accuracy (%)	Number of correct answers per person	Mean reading time (ms)	Mean answering time (ms)
Comparative	82	3–10	729.0	3924.9
HA	74	4–10	766.8	3142.4
LA	63	4–10	760.7	3075.3
Locative	87	3–10	676.7	4193.9
ORC	86	4–10	674.6	3328.4
Temporal	91	5–10	660.3	3239.9
Control	93	32–40	595.6	2934.2

**Table 4:** Significant model outputs for accuracy analysis in Experiment 1.

Comparison	Model output
Comparative vs. Control	β = −1.73, SE = 0.33, z = −5.25, *p* < 0.001***
HA vs. Control	β = −2.05, SE = 0.31, z = −6.49, *p* < 0.001***
LA vs. Control	β = −2.78, SE = 0.31, z = −8.91, *p* < 0.001***
LA vs. Locative	β = −1.59, SE = 0.38, z = −4.20, p < 0.001***
Locative vs. Control	β = −1.19, SE = 0.33, z = −3.61, *p* = 0.004**
ORC vs. Control	β = −1.36, SE = 0.33, z = −4.18, *p* < 0.001***
ORC vs. LA	β = 1.42, SE = 0.37, z = 3.78, *p* = 0.002**
Temporal vs. HA	β = 1.45, SE = 0.40, z = 3.63, *p* = 0.004**
Temporal vs. LA	β = 2.18, SE = 0.39, z = 5.50, p < 0.001***

* indicate results significant at the *p*<0.05 level, ** indicate results significant at the *p*<0.01 level, and *** to indicate results significant at the *p*<0.001 level.

In high and low attachment examples, the source of processing difficulties is morphosyntactic, while in comparative examples, it is in the syntax-semantics mapping. We believe that comparative sentences were more difficult than two other sentence types with syntax-semantics mapping problems (locative and temporal) because three rather than two items had to be ordered. As for morphosyntactic problems, our results and a series of dedicated experiments in (Chernova, 2015; Chernova et al., 2016; Slioussar et al., 2022) show that several factors contribute to processing complexity: LA is more difficult than HA, retrieving case features is more difficult than number and gender features. Finally, all such examples contain a participial construction, which adds to the syntactic complexity of the sentence. In other words, having only one potentially difficult structure or complex operation is not enough to cause significant processing difficulties in adult native speakers.

Object relative clause sentences also illustrate this point. They have an embedded relative clause and a noncanonical word order. Our pilot study showed that having only one of these properties does not cause significant processing problems in healthy adult native speakers.

Word-by-word reading times and answering times also revealed some interesting differences between the target constructions. Word-by-word reading times correlate with accuracy (see Table 5). High and low attachment constructions and comparative constructions (the ones that had the lowest accuracy) had significantly longer word-by-word reading times than control sentences and other target sentence types, which did not differ from each other. Temporal constructions with the highest accuracy rate had the shortest average word reading time.

**Table 5:** Significant model outputs for word reading time analysis in Experiment 1.

Comparison	Model output
Comparative vs. Control	β = 204.19, SE = 19.83, z = 10.30, *p* < 0.001***
HA vs. Control	β = 178.84, SE = 19.67, z = 9.09, *p* < 0.001***
LA vs. Control	β = 187.29, SE = 19.67, z = 9.52, *p* < 0.001***
LA vs. Locative	β = 172.12, SE = 24.86, z = 6.92, *p* < 0.001***
Locative vs. Comparative	β = −189.02, SE = 24.98, z = −7.56, *p* < 0.001***
Locative vs. HA	β = −163.68, SE = 24.85, z = −6.58, *p* < 0.001***
ORC vs. Comparative	β = −183.07, SE = 24.5, z = −7.47, *p* < 0.001***
ORC vs. HA	β = −157.72, SE = 24.36, z = −6.47, *p* < 0.001***
ORC vs. LA	β = −166.17, SE = 24.37, z = −6.82, p < 0.001***
Temporal vs. Comparative	β = −219.18, SE = 24.68, z = −8.88, *p* < 0.001***
Temporal vs. HA	β = −193.83, SE = 24.55, z = −7.90, *p* < 0.001***
Temporal vs. LA	β = −202.28, SE = 24.55, z = −8.24, *p* < 0.001***

* indicate results significant at the *p*<0.05 level, ** indicate results significant at the *p*<0.01 level, and *** to indicate results significant at the *p*<0.001 level.

Answering times present a different picture (see Table 6). Two construction types with the lowest accuracy, low and high attachment sentences, had the shortest answering times. Temporal constructions that have the highest accuracy come third. The longest answering times were registered for locative and comparative constructions (most pairwise comparisons with the other conditions were significant), i.e., comparative constructions have relatively low accuracy, long word reading times and answering times; the opposite is true for temporal constructions; while for low and high attachment sentences, these measures do not go hand in hand.

**Table 6:** Significant model outputs for question answering time analysis in Experiment 1.

Comparison	Model output
Comparative vs. Control	β = 1508.02, SE = 164.46, z = 9.17, *p* < 0.001***
HA vs. Comparative	β = −1305.06, SE = 205.58, z = −6.35, *p* < 0.001***
LA vs. Comparative	β = −1425.89, SE = 205.63, z = −6.93, *p* < 0.001***
LA vs. Locative	β = −1362.73, SE = 201.76, z = −6.75, *p* < 0.001***
Locative vs. Control	β = 1444.87, SE = 159.55, z = 9.056, *p* < 0.001***
Locative vs. HA	β = 1241.91, SE = 201.69, z = 6.16, *p* < 0.001***
ORC vs. Comparative	β = −1187.35, SE = 205.29, z = −5.79, *p* < 0.001***
ORC vs. Locative	β = −1124.19, SE = 201.41, z = −5.58, *p* < 0.001***
Temporal vs. Comparative	β = −1273.10, SE = 204.98, z = −6.21, *p* < 0.001***
Temporal vs. Locative	β = −1209.94, SE = 201.08, z = −6.02, *p* < 0.001***

* indicate results significant at the *p*<0.05 level, ** indicate results significant at the *p*<0.01 level, and *** to indicate results significant at the *p*<0.001 level.

This may point to two different manifestations of processing complexity. In some cases, arriving at any coherent interpretation is difficult (mapping syntax and semantics in comparative constructions). In the other cases, one arrives at some interpretation easily, but it is often not the correct one (retrieving a wrong case feature in high and low attachment sentences).

In total, the results of Experiment 1 demonstrate that the SCT is a valid tool to assess syntactic processing abilities, which does not suffer from ceiling effects, captures individual variation and reflects the relationship between syntactic processing skills and working memory span that was observed in the previous studies.

## 5. Experiment 2: A reading and listening study

In Experiment 2, we aimed to replicate the results of Experiment 1, which is crucial to ensure the reliability of the SCT, and at the same time to study the role of the presentation modality, written or oral. We expect the effects of syntactic complexity to be same in the reading and listening modes reflecting the supramodal nature of syntactic processing (Constable et al., 2004).

### 5.1. Participants

Ninety-eight native speakers of Russian (50 female) aged 19–53 (mean age 38) volunteered to take part in Experiment 2. The participants were recruited *via* the crowdsourcing platform Yandex. Toloka and were paid 3$ for participation. All of them reported that they had no language disorders or neurological problems. No participant took part in Experiment 1 or in the pilot study.

### 5.2. Materials

The materials were the same as in the Experiment 1. For the oral part of the experiment, all sentences were recorded by a male native Russian speaker who spoke with a natural and consistent pace and volume and was unaware of the purpose of the study.

### 5.3. Procedure

Experiment 2 was conducted online on the PCIbex platform (Zehr and Schwarz, 2018). We divided our experimental materials in two halves, making sure that both halves have the same number of control and of target sentences in different conditions and are minimally different in terms of sentence length. The experiment had a within-subject design, so there were four experimental lists. In the first list, the first half of the sentences was presented in the listening mode and then the second half in the reading mode. In the second list, the second half was presented in the listening mode and then the first half in the reading mode. The third and fourth lists included the same materials as the first and the second respectively, but the reading part preceded the listening one.

The participants were asked to read sentences in a self-paced reading mode or to listen to them and answer the questions as quickly and accurately as they could. After every sentence, they answered a question by choosing one of the response options. The self-paced reading procedure was described in Section 4.3. As for the listening task, an audiofile with a sentence was played when the participant clicked with a right button of mouse. It was possible to listen to every sentence only once.

### 5.4. Analysis

We analyzed participants’ question answering time and accuracy. Word-by-word reading times were not analyzed because they are irrelevant for the listening mode. Answering times that exceeded a threshold of 2.5 standard deviations by condition were excluded (Ratcliff, 1993). In total, 9% of the data were excluded. The methods of the statistical analysis were the same as in Experiment 1.

### 5.5. Results and discussion

First of all, we analyzed accuracy using two factors: whether the sentence was a target or a control and modality. Target sentences were more difficult to process than controls in both modalities (listening mode: 81.3 vs. 97.1% correct answers on average; reading mode: 75.5 vs. 94.4% correct answers on average). The target/control factor was significant (*β* = −2.03, SE = 0.25, *t* = −8.25, *p* < 0.001), while the modality factor and the interaction between the two factors were not, although we can observe the tendency for lower accuracy in the reading mode, especially in stimulus sentences. Thus, the SCT can be administered both in the reading and in the listening mode: the difference between target and control sentences is found in both modalities. Similar results for different target sentence types presented below further stress the validity of this conclusion.

Then we analyzed question answering times in the same way. Target sentences had longer answering times in both modalities (listening mode: 4639.0 vs. 4261.4 ms on average; reading mode: 3615.1 vs. 3066.1 ms on average), and it took significantly more time to answer the questions in listening mode compared to reading mode (4,585 vs. 3,536 ms on average). The target/control factor (*β* = 1176.3, SE = 119.9, *t* = 9.82, *p* < 0.001), the modality factor (*β* = −699.3, SE = 160.5, *t* = −4.36, *p* < 0.001) and their interaction (*β* = −283.3, SE = 111.2, *t* = −2.55, *p* = 0.011) reached significance, indicating that the difference between the two modalities was more pronounced for target sentences than for controls. The effect of modality could be caused by our procedure: the questions and answers were always presented in the reading mode, so increased response times may reflect the modality switch cost.

**Figure 2:**
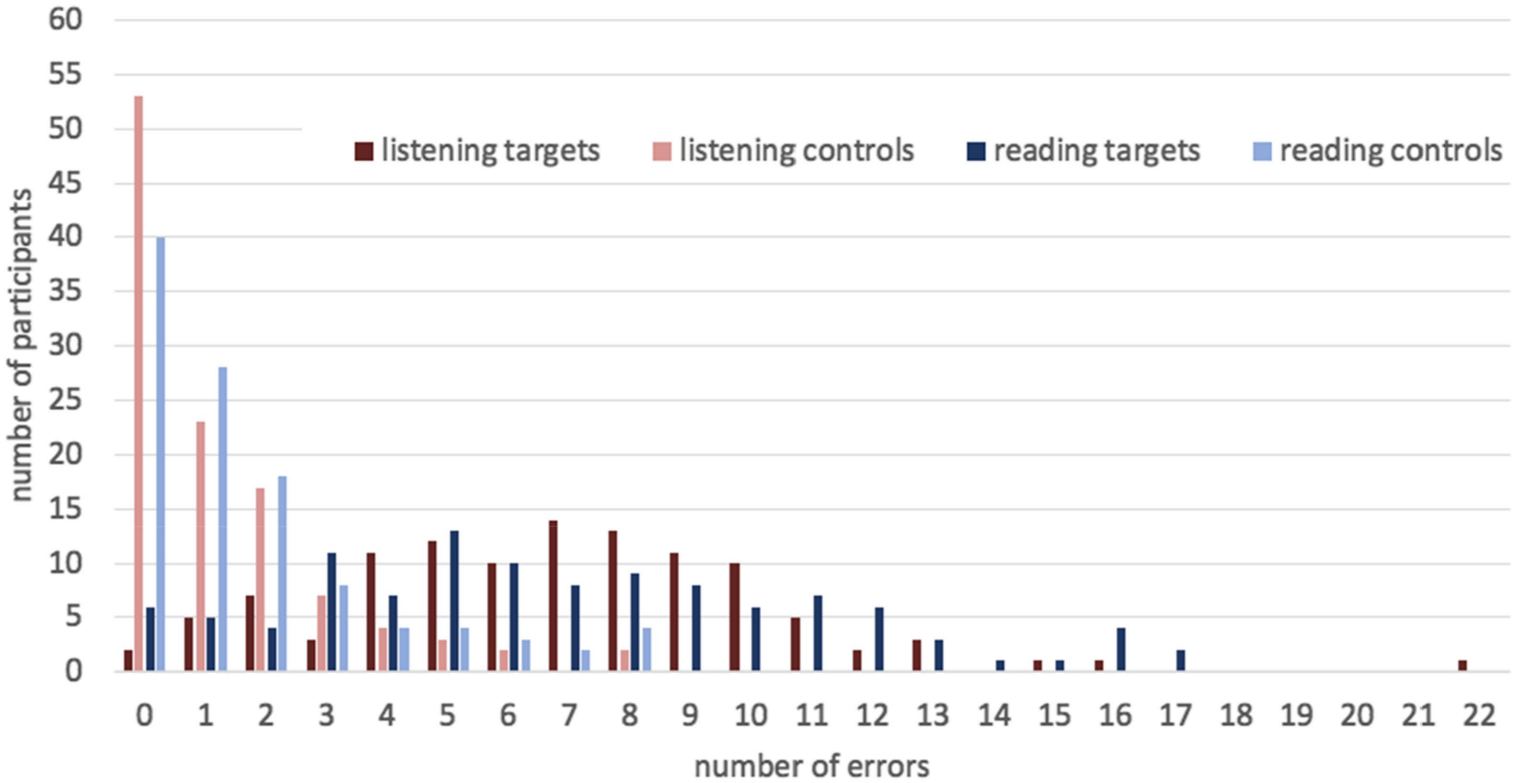
The number of errors per participant in target and control sentences in Experiment 2.

Like in Experiment 1, there was variation in accuracy between participants (see Figure 2). In the listening mode, they made from 0 to 21 errors in target sentences, which means from 100 to 30% correct answers. The mean number of errors was 6.9, SD = 3.6. In the reading mode, they made 0–17 errors, i.e., gave 100–43% correct answers. The mean number of errors was 7.0, SD = 4.2. The variation in control sentences was less noticeable. In both modes, participants made 0–8 errors, i.e., 100–60% correct answers.^11^ The mean number of errors was 1.3, SD = 1.9 in the listening mode and 1.7, SD = 2.2 in the reading mode.

Now let us look at different construction types. Average accuracy and question answering times are presented in Table 7. Like in Experiment 1, accuracy rates for every target construction, except for the temporal one, were significantly different from controls both in the reading and in the listening mode (see Table 8). Other results from Experiment 1 were also replicated in both modalities. Namely, low attachment sentences were the most difficult to interpret, being significantly different from all other target sentence types, while temporal sentences were the easiest (in the listening mode, they were significantly different from all other target sentence types, while in the reading mode, in which average accuracy for all target sentence types was lower, only some differences reached significance). If we assume that syntactic processing relies on the same mechanisms in both modalities (see Constable et al., 2004; Shankweiler et al., 2008; Braze et al., 2011), such parallels are expected (Table 9).

**Table 7:** Average question answering times and accuracy in the reading and listening modes in Experiment 2.

Sentence type	Listening: average accuracy (%)	Number of correct answers per person	Mean answering time (ms)	Reading: average accuracy (%)	Number of correct answers per person	Mean answering time (ms)
Comparative	89	1–5	5596.4	77	1–5	3917.3
HA	78	0–5	4434.6	84	0–5	3330.8
LA	47	0–5	4352.2	50	0–5	3545.2
Locative	89	0–5	4590.3	78	1–5	3843.2
ORC	89	1–5	4524.6	79	1–5	3696.4
Temporal	96	1–5	4335.7	86	1–5	3557.6
Control	97	12–20	4261.4	94	12–20	3066.1

**Table 8:** Significant model outputs for accuracy analysis in Experiment 2.

Comparison	Listening: model output	Reading: model output
Control vs. Comparative	*β* = 1.39, SE = 0.19, *z* = 7.20, *p* < 0.001***	*β* = 0.83, SE = 0.20, *z* = 4.06, *p* < 0.001***
HA vs. Control	*β* = −1.28, SE = 0.16, *z* = −7.83, *p* < 0.001***	*β* = −1.17, SE = 0.19, *z* = −6.08, *p* < 0.001***
LA vs. Comparative	*β* = −2.00, SE = 0.20, *z* = −10.02, *p* < 0.001***	*β* = −2.23, SE = 0.22, *z* = −9.97, *p* < 0.001***
LA vs. HA	*β* = −2.12, SE = 0.17, *z* = −12.13, *p* < 0.001***	*β* = −1.92, SE = 0.23, *z* = −8.45, *p* < 0.001***
LA vs. Locative	*β* = −2.43, SE = 0.20, *z* = −12.36, *p* < 0.001***	*β* = −2.23, SE = 0.22, *z* = −9.95, *p* < 0.001***
LA vs. Control	*β* = −3.47, SE = 0.15, *z* = −22.65, *p* < 0.001***	*β* = −3.08, SE = 0.17, *z* = −18.22, *p* < 0.001***
Locative vs. Control	*β* = −0.99, SE = 0.19, *z* = −5.29, *p* < 0.001***	*β* = −0.83, SE = 0.18, *z* = −4.52, *p* < 0.001***
ORC vs. LA	*β* = 2.86, SE = 0.20, *z* = 13.63, *p* < 0.001***	*β* = 1.80, SE = 0.22, *z* = 8.37, *p* < 0.001***
ORC vs. Control	*β* = −0.82, SE = 0.18, *z* = −4.59, *p* < 0.001***	*β* = −1.27, SE = 0.18, *z* = −6.99, *p* < 0.001***
Temporal vs. Comparative	*β* = 1.00, SE = 0.25, *z* = 4.89, *p* < 0.001***	*β* = 0.38, SE = 0.26, *z* = 1.45, *p* = 0.835
Temporal vs. HA	*β* = 1.10, SE = 0.23, *z* = 4.85, *p* < 0.001***	*β* = 0.68, SE = 0.25, *z* = 2.67, *p* = 0.069
Temporal vs. LA	*β* = 3.12, SE = 0.22, *z* = 14.6, *p* < 0.001***	*β* = 2.60, SE = 0.24, *z* = 10.70, *p* < 0.001***
Temporal vs. Locative	*β* = 0.79, SE = 0.24, *z* = 3.24, *p* = 0.001**	*β* = 0.37, SE = 0.25, *z* = 1.48, *p* = 0.835
Temporal vs. ORC	*β* = 0.63, SE = 0.21, *z* = 2.69, *p* = 0.050*	*β* = 0.78, SE = 0.25, *z* = 3.21, *p* = 0.013*

* indicate results significant at the *p*<0.05 level, ** indicate results significant at the *p*<0.01 level, and *** to indicate results significant at the *p*<0.001 level.

**Table 9:** Significant model outputs for answering time analysis in Experiment 2.

Comparison	Listening: model output	Reading: model output
Control vs. Comparative	*β* = −2203.6, SE = 107.4, *z* = −20.52, *p* < 0.001***	*β* = −1791.7, SE = 109.2, *z* = −16.41, *p* < 0.001***
HA vs. Comparative	*β* = −1765.1, SE = 135.8, *z* = −13.00, *p* < 0.001***	*β* = −1363.3, SE = 139.9, *z* = −9.74, *p* < 0.001***
HA vs. Control	*β* = 855.9, SE = 86.1, *z* = 9.94, *p* < 0.001***	*β* = 323.9, SE = 109.4, *z* = 2.96, *p* = 0.015*
LA vs. Comparative	*β* = −1261.6, SE = 137.8, *z* = −9.16, *p* < 0.001***	*β* = −1168.3, SE = 140.8, *z* = −8.23, *p* < 0.001***
LA vs. HA	*β* = 503.5, SE = 145.0, *z* = 3.47, *p* = 0.003**	*β* = 194.9, SE = 121.5, *z* = 1.60, *p* = 0.326
LA vs. Locative	*β* = −671.2, SE = 137.2, *z* = −4.89, *p* < 0.001***	*β* = −373.9, SE = 127.4, *z* = −2.93, *p* = 0.023*
LA vs. Control	*β* = 1055.3, SE = 87.9, *z* = 12.0, *p* < 0.001***	*β* = 561.1, SE = 96.3, *z* = 5.83, *p* < 0.001***
Locative vs. Comparative	*β* = −590.4, SE = 138.0, *z* = −4.28, *p* < 0.001***	*β* = −794.4, SE = 144.6, *z* = −5.49, *p* < 0.001***
Locative vs. HA	*β* = 1174.8, SE = 141.0, *z* = 8.33, *p* < 0.001***	*β* = 568.9, SE = 125.9, *z* = 4.52, *p* < 0.001***
Locative vs. Control	*β* = 1403.9, SE = 92.8, *z* = 15.13, *p* < 0.001***	*β* = 1252.0, SE = 92.8, *z* = 13.49, *p* < 0.001***
ORC vs. Comparative	*β* = −940.0, SE = 135.0, *z* = −6.96, *p* < 0.001***	*β* = −1072.0, SE = 139.2, *z* = −7.71, *p* < 0.001***
ORC vs. HA	*β* = 825.2, SE = 144.5, *z* = 5.71, *p* < 0.001***	*β* = 291.3, SE = 119.2, *z* = 2.44, *p* = 0.073
ORC vs. Control	*β* = 1143.9, SE = 85.2, *z* = 13.42, *p* < 0.001***	*β* = 804.5, SE = 96.6, *z* = 8.33, *p* < 0.001***
Temporal vs. Comparative	*β* = −1447.0, SE = 145.6, *z* = −9.94, *p* < 0.001***	*β* = −1520.6, SE = 138.6, *z* = −10.97, *p* < 0.001***
Temporal vs. LA	*β* = −185.4, SE = 143.5, *z* = −1.29, *p* = 0.196	*β* = −352.3, SE = 119.9, *z* = −2.94, *p* = 0.023*
Temporal vs. Locative	*β* = −856.6, SE = 140.7, *z* = −6.09, *p* < 0.001***	*β* = −726.2, SE = 124.0, *z* = −5.90, *p* < 0.001***
Temporal vs. ORC	*β* = −507.0, SE = 140.8, *z* = −3.60, *p* = 0.002**	*β* = −448.6, SE = 117.4, *z* = −3.82, *p* = 0.001**
Temporal vs. Control	*β* = 685.1, SE = 84.2, *z* = 8.14, *p* < 0.001***	*β* = 251.8, SE = 92.2, *z* = 2.73, *p* = 0.031*

* indicate results significant at the *p*<0.05 level, ** indicate results significant at the *p*<0.01 level, and *** to indicate results significant at the *p*<0.001 level.

Important parallels between Experiments 1 and 2 and between the two modalities can also be found in answering times. They were the longest for comparative and locative constructions (for comparative constructions, all pairwise comparisons with other target sentence types were significant; for locative, the difference with object relative clauses did not reach significance in both modalities). The shortest answering times were registered for high and low attachment and temporal constructions. It is also important to note that all target sentence types were significantly different from controls in all modalities.

## 6. Conclusion

In this paper, we presented the Sentence Comprehension Test (SCT) designed to measure syntactic processing abilities in healthy adult native speakers of Russian. Syntactic processing abilities are a crucial part of the linguistic competence. Thus, it is not surprising that their assessment is included in many tests targeting first and second language acquisition, developmental and acquired language disorders. However, only two previous studies (Acheson et al., 2008; Dąbrowska, 2018), both of them on English, tried to develop a similar test for healthy adult native speakers (Dąbrowska also tested L2 participants). It is not the case that such speakers always show excellent performance in syntactic processing tasks—quite on the contrary, considerable variability was detected in many studies (e.g., Farmer et al., 2012). The problem is that this variability can be observed when participants’ attention is distracted from syntactic processing. When native speakers focus on it—which does not happen normally, but is hard to avoid in a dedicated test—they tend to reach ceiling performance.

Therefore, our goal when creating the SCT was to select target syntactic constructions that would be difficult enough and diverse enough and to choose the right comprehension questions and target-control ratio so that these problems could be avoided. Experiments 1 and 2 demonstrated that we succeeded. The SCT works effectively both in the reading and in the listening mode: it does not show ceiling effects and captures inter-speaker variation. In most previous studies, working memory span was mentioned as the most important factor defining this variation (e.g., Daneman and Carpenter, 1980; King and Just, 1991; MacDonald et al., 1992; Caplan and Waters, 1999, 2013; Swets et al., 2008; Caplan et al., 2013). In Experiment 1, we found a significant correlation between individual accuracy rates for target sentences in the SCT and working memory test scores.

Based on previous studies on Russian and other languages, we selected ten target constructions for the pilot version of the SCT, and then six constructions were chosen for the final version: object relative clauses, sentences with a high or low attachment of a participial clause to a complex noun phrase (HA and LA constructions), temporal, locative and comparative constructions. In the first three sentence types, the sources of processing difficulties are syntactic or morphosyntactic, in the last three, they lie at the intersection between syntax and semantics, i.e., the selected constructions are diverse enough. Accuracy rates for all target sentence types were lower than for control sentences of comparable length in two different experiments in the reading and listening modality, and these differences were significant for all constructions, except for temporal ones. Notably, all constructions we selected were different from the ones used by Acheson et al. (2008) and Dąbrowska (2018), except for object relative clauses.

We measured not only comprehension accuracy, but also word-by-word reading times and question answering times. Similar results were obtained in Experiments 1 and 2 and in different modalities. LA sentences had the lowest accuracy rates and the longest word-by-word reading times, while temporal sentences had the highest accuracy rates and the shortest word-by-word reading times. Comparative and locative constructions had the longest, and HA/LA and temporal constructions the shortest answering times. In general, word-by-word reading times tend to correlate with accuracy, while question answering times present a different picture, which may point to different manifestations of processing difficulty. In some cases, arriving at any coherent interpretation is difficult (mapping syntax and semantics in comparative constructions). In the other cases, one arrives at some interpretation easily, but it is often not the correct one (retrieving a wrong case feature in high and low attachment sentences).

The question whether listening comprehension is more or less costly than reading comprehension is debated in the literature, but there is a general agreement that the same syntactic processing system is used in both modalities. Experiment 2 showed that the average accuracy was slightly lower in the reading mode, while the question answering times are significantly longer in the listening mode. At the same time, as we noted above, the most important results were replicated in both modalities. This confirms the reliability of the SCT and gives more freedom to its potential users.

To conclude, the test can be used in various psycholinguistic and neurolinguistic studies to assess individual differences in sentence processing skills. Moreover, our next goal is to adapt it for L2 speakers of advanced levels.

As for the possible limitations of the current study, we can note that our participant samples do not represent the population of Russian speakers as a whole. We recruited university students in Experiment 1 and people subscribed to the Yandex. Toloka crowdsourcing platform in Experiment 2. Unfortunately, this problem plagues many experimental linguistic studies, although the few exceptions show that recruiting participants with a more diverse background may be extremely rewarding (e.g., Dąbrowska, 2012). Therefore, we would be interested to conduct further research on sociolinguistic aspects of sentence comprehension: the effects of age, educational level, profession etc. At the same time, the results of Experiment 2 with a more diverse sample of participants are similar to those of Experiment 1, and we do not see any increase in individual variability range. So we believe that recruiting an even more diverse pool of participants will not change the general conclusions we reached in this study.

Another very promising direction for further research are cross-linguistic comparisons. We saw that certain constructions, like object relative clauses, tend to cause processing difficulties in different languages. But some sources of syntactic complexity are language-specific, for example, connected to processing of rich morphology in one language and to processing of ambiguity caused by scarce morphology in the other. It would be extremely interesting to explore these differences. Such comparisons can be made when tools like Sentence Comprehension Test appear for typologically different languages.

## Data availability statement

The raw data supporting the conclusions of this article will be made available by the authors, without undue reservation.

## Ethics statement

Ethical review and approval was not required for the study on human participants in accordance with the local legislation and institutional requirements. The patients/participants provided their written informed consent to participate in this study.

## Author contributions

DC: general idea of the study, data collection, data analysis, interpretation, and drafting the manuscript. AN: data collection, data analysis, and drafting the manuscript. NS: general idea of the study, overseeing data analysis, critical revision, and editing of the manuscript. All authors contributed to the article and approved the submitted version.

## Funding

The study was supported by grant no. 21-78-00064 from RSF, https://rscf.ru/project/21-78-00064/

## Conflict of interest

The authors declare that the research was conducted in the absence of any commercial or financial relationships that could be construed as a potential conflict of interest.

## Publisher’s note

All claims expressed in this article are solely those of the authors and do not necessarily represent those of their affiliated organizations, or those of the publisher, the editors and the reviewers. Any product that may be evaluated in this article, or claim that may be made by its manufacturer, is not guaranteed or endorsed by the publisher.

## Footnotes

^1^Some authors use different tests to assess different working memory components (e.g., Boyle et al., 2013), while the others do not specify which working memory model they rely on and which component their test is assumed to target. In particular, this is the case for Daneman and Carpenter’s (1980) test whose adaptation we used in our study.

^2^Here and below we use the following abbreviations: NOM, nominative case, ACC, accusative case, GEN, genitive case, DAT, dative case, INS, instrumental case, LOC, locative case, SG, singular, PL, plural, M, masculine, F, feminine, 1, 1st person, 2, 2nd person, 3, 3rd person, REFL, reflexive, ADJ, adjective, PART, participle.

^3^If the head noun and the dependent noun differ in number, gender or case, the form of the participle will unambiguously indicate the attachment site, unless case syncretism interferes (the forms of Russian adjectives and participles coincide in some cases). We did not include sentences with syncretic participle forms in our study.

^4^Another piece of evidence comes from the study reported by Antropova et al. (2022) who studied examples with number and case agreement errors on participial modifiers [all sentences discussed in the present paper and in Chernova, 2015, Chernova et al., 2016, Slioussar et al., 2022 are grammatical]. Antropova et al. demonstrated that such errors cause an immediate significant delay in reading times, which means that they are efficiently detected.

^5^Passives do not follow the ‘agent first’ strategy, i.e., the first noun phrase mentioned in the sentence does not have the agent semantic role, corresponding to the object of a transitive verb, so passives are often included in the noncanonical word order group.

^6^ ^Russian has flexible word order, so many orders that are ungrammatical in English are possible in Russian.^

^7^www.r-project.org

^8^ORC stimuli were kindly provided by the authors of (Malyutina et al., 2018).

^9^This test is usually carried out in person, but we had to conduct it online due to pandemic restrictions.

^10^www.r-project.org

^11^The variation was greater than in Experiment 1, but only due to several participants: 92 participants (94%) made two or fewer errors with control sentences in the listening mode and three or fewer errors in the reading mode.
